# Supplementary material for: Sfrp1 deficiency makes retinal photoreceptors prone to degeneration
Source: Sci Rep. 2020 Mar 20;10:5115. doi: 10.1038/s41598-020-61970-8 (PMC7083943; doi:10.1038/s41598-020-61970-8)
Supplement: Supplementary file 1 — Supplementary information [file 41598_2020_61970_MOESM1_ESM.pdf]

Sfrp1 deficiency makes retinal photoreceptors prone to degeneration

Elsa Cisneros*, Fabiana di Marco*, Javier Rueda-Carrasco, Concepción Lillo, Guadalupe Pereyra, María Jesús Martín-Bermejo, Alba Vargas, Rocío Sanchez, África Sandonís, Pilar Esteve and Paola Bovolenta

Supplementary Information

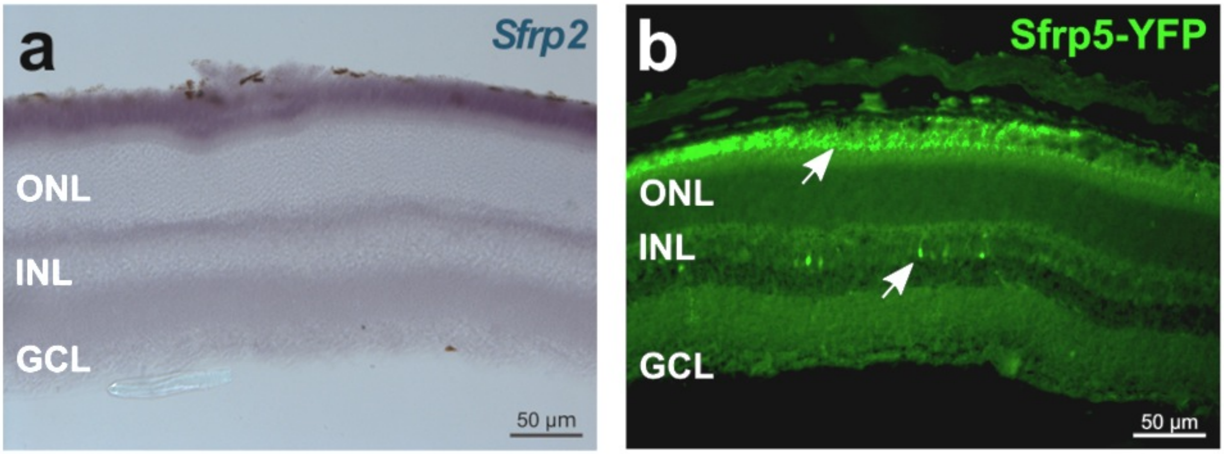

Figure S1. Extended information from Figure 1. a) Frontal cryostat section from 1 month-old wt animal hybridized with a probe for *Sfrp2*. No specific signal was observed. b) Frontal cryostat section from 1 month-old *Sfrp5*^{tm1Aksh} animal immunostained for YFP reporter distribution. Note the presence of fluorescent signal in the RPE and sparse cells of the INL.

a

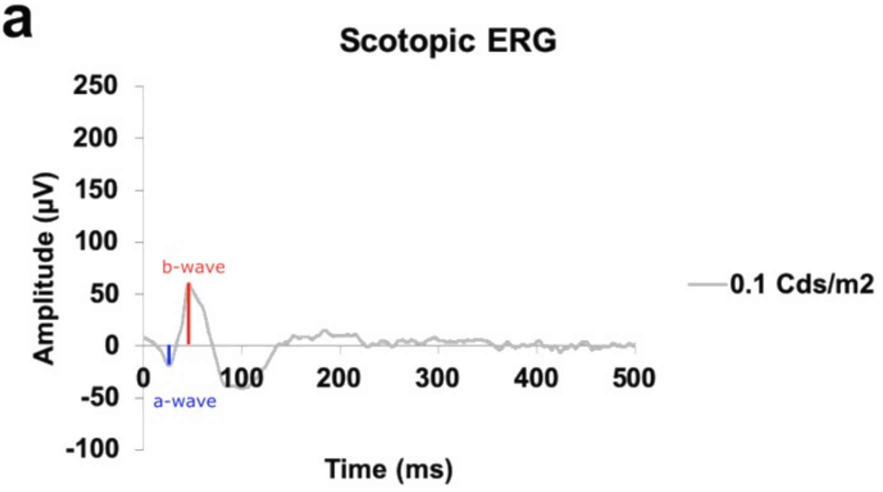

Figure S2. Extended information from Figure 2. Typical recording under scotopic conditions were a (blue) and b wave amplitudes (red) have been indicated.

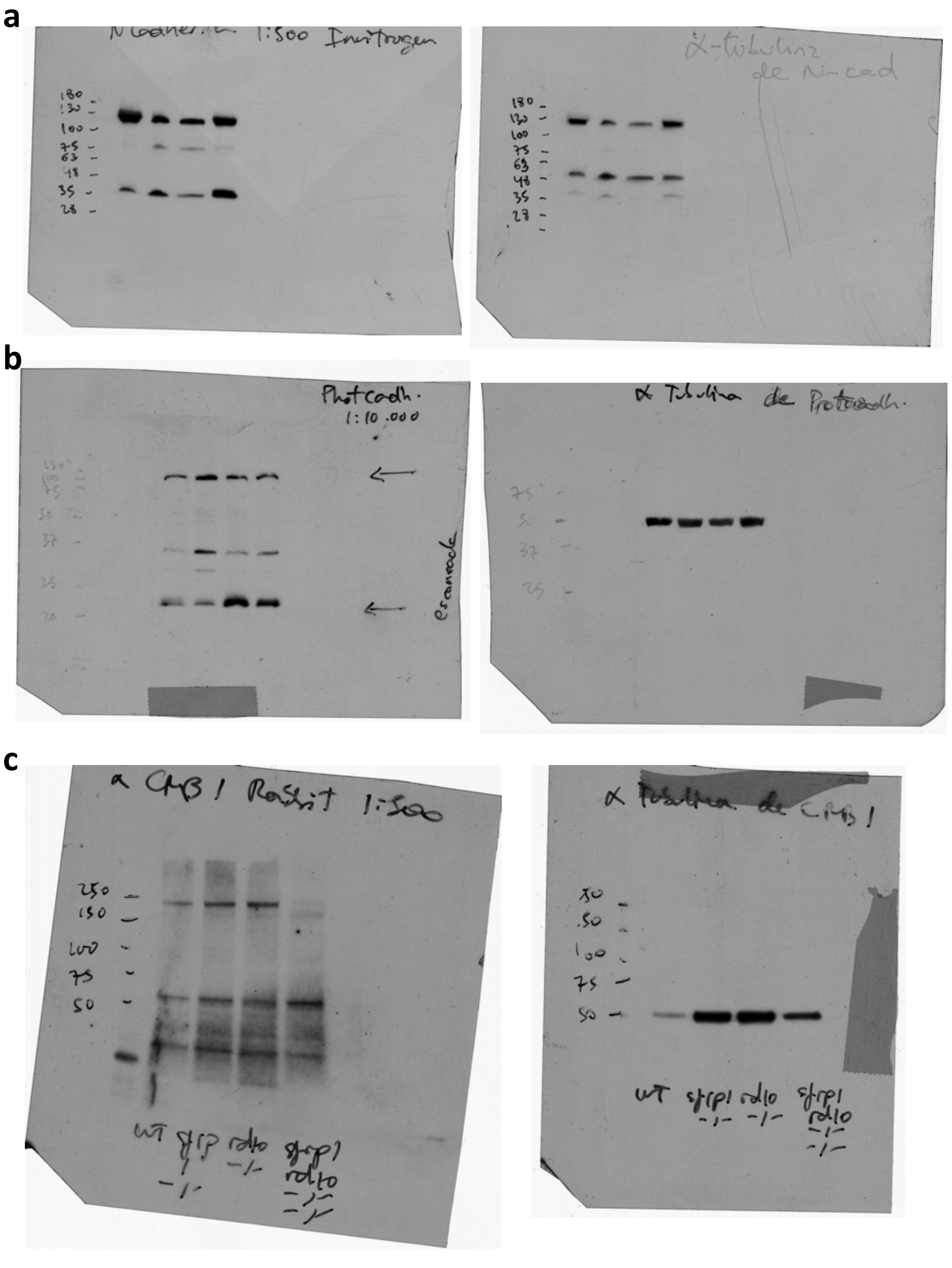

Figure S3. Extended information from Figure 5. a-c) Uncropped Western Blot images displayed in Fig. 5c, d, e (left) respectively together with their relative controls (right).
